# The protection afforded by kefir against cyclophosphamide induced testicular toxicity in rats by oxidant antioxidant and histopathological evaluations

**DOI:** 10.1038/s41598-024-67982-y

**Published:** 2024-08-09

**Authors:** Songul Cetik Yildiz, Cemil Demir, Mustafa Cengiz, Halit Irmak, Betul Peker Cengiz, Adnan Ayhanci

**Affiliations:** 1 Department of Medical Services and Techniques, Health Services Vocational School, Mardin Artuklu University, Mardin Artuklu University Campus, 47200 Mardin, Turkey; 2 Department of Elementary Education, Faculty of Education, Siirt University, Siirt, Turkey; 3 Department of Computer Sciences, Mardin Artuklu University, Mardin, Turkey; 4 Eskisehir Yunus Emre State Hospital, Eskisehir, Turkey; 5 Department of Biology, Science Faculty, Eskisehir Osmangazi University, Eskişehir, Turkey

**Keywords:** Cyclophosphamide, Testicular toxicity, Oxidative stress, Kefir, Antioxidant, Rats, Diseases, Cancer prevention, Immunotherapy

## Abstract

Cyclophosphamide (CTX) is the most commonly used effective alkylating drug in cancer treatment, but its use is restricted because its toxic side effect causes testicular toxicity. CTX disrupts the tissue redox and antioxidant balance and the resulting tissue damage causes oxidative stress. In our study based on this problem, kefir against CTX-induced oxidative stress and testicular toxicity were investigated. Rats were divided into 6 groups: control, 150 mg/kg CTX, 5 and 10 mg/kg kefir, 5 and 10 mg/kg kefir + 150 CTX. While the fermented kefirs were mixed and given to the rats for 12 days, CTX was given as a single dose on the 12th day of the experiment. Testis was scored according to spermatid density, giant cell formation, cells shed into tubules, maturation disorder, and atrophy. According to our biochemical findings, the high levels of total oxidant status (TOS), and the low levels of total antioxidant status (TAS) in the CTX group, which are oxidative stress markers, indicate the toxic effect of CTX, while the decrease in TOS levels and the increase in TAS levels in the kefir groups indicate the protective effect of kefir. In the CTX-administered group, tubules with impaired maturation and no spermatids were observed in the transverse section of the testicle, while in the kefir groups, the presence of near-normal tubule structures and tubule lumens despite CTX showed the protective effect of kefir. In our study, it was observed that kefir had a protective and curative effect on CTX-induced toxicity and oxidative stress and could be a strong protector.

## Introduction

Antineoplastic drugs may have gonadotoxic effects in varying amounts depending on factors such as the dose, type, and duration of the drug used. Cyclophosphamide (CTX), one of these cytotoxic agents, can cause infertility due to permanent and long-term gonadal toxicity^1,2^. Although the cytotoxic effects of CTX, which is widely used in cancer chemotherapy, limit the use of the drug, it also increases oxidative stress, which mediates the disruption of redox balance after exposure and causes many biochemical and physiological disorders^2^. In order for CTX to exert its potential coccoidal effect, it must first be metabolized and activated. CTX-induced immunosuppression occurs due to the release of its metabolites rather than the drug itself. The metabolism of CTX in the liver the formation of acrolein, a cytotoxic metabolite, and a simultaneous increase in reactive oxygen species (ROS) and lipid peroxidation are associated with oxidative stress^3^. The alkylating metabolite of CTX, phosphoramide mustard, is responsible for therapeutic activity and produces a wide range of adverse effects, such as testicular toxicity. Acrolein has also been reported to have adverse effects on fertility, including hemorrhagic cystitis and apoptotic changes in the testicles^4^. Furthermore, because the spermatozoa's mitochondrial membrane is rich in polyunsaturated fatty acids and deficient in antioxidants, it is more vulnerable to lipid peroxidation^5^. In the germinal epithelium, spermatogenesis is a vigorous meiotic division cycle that requires a lot of oxygen from the mitochondria. Nonetheless, low oxygen tension results from inadequate testicular vascularization. Because Leydig cells are sensitive to oxidative stress in both spermatogenesis and steroidogenesis, low oxygen levels may shield tissues from damage by free radicals^6^. These may explain how CTX causes toxicity in organs such as testicles, as CTX disrupts tissue redox balance, and tissue damage resulting from this disruption causes oxidative stress^7^. In many studies, It has been reported that CTX was histologically in testicular tubules may cause a decrease in germinal epithelium height and seminiferous tubule diameter, tubular atrophy, disruption of germinal epithelium and basement membrane integrity, edema in the interstitium, increase in collagen density and Leydig cell atrophy^8^. Normally, free radicals occur in the mitochondria of testicular cells but are scavenged by the antioxidant defense system^5,9^. Kefir, a natural antioxidant, is the most important prebiotic and probiotic fermented milk product needed to prevent oxidative damage and cytotoxicity caused by CTX. Fermented kefir slows the growth of cancer cells and accelerates apoptosis, with its immunotherapeutic, antioxidant, and antitumor properties^10^. Studies have shown that kefir exhibits activities such as antioxidative, antimicrobial, and anticarcinogenic properties, and protection against apoptosis^11^.

## Results

In this experimental study, the possible protective effect of kefir in the testicular damage model created with cyclophosphamide (CTX) was examined with biochemical and histopathological parameters, and the antioxidant and cytoprotective effects of kefir on testicular toxicity were compared. Since the kefir we used in our experimental study created microbial flora at different times, fermented kefirs on different days were tested. As a result of the test we conducted on the kefirs on different days, no significant change was observed between the kefirs of the 1st, 2nd, and 3rd days, so the kefirs of all days were mixed and used to be given to the rats. Kefir used in experimental studies was used in very different doses and durations. In our study, we gave kefir to rats by gavage method for 12 days.

As seen in Table 1, our data were scored according to testicular spermatid density, giant cell formation, tubule-sloughed cells, maturation disorder, and atrophy. In the CTX-administered group, testicular spermatid density, giant cell formation, cells sloughed into tubules, maturation disorder, and atrophy levels were seen as moderate changes (score 2). In the groups given CTX + kefir, this moderate change decreased to a slight change (score 1) and approached the control group (Table 1).

**Table 1:** Scoring based on testicular spermatid density, giant cell formation, tubule-sloughed-cells, maturation disorder and atrophy.

Groups	Testicular Score
Control	0^a^
150 mg/kg CTX	2^c^
5 mg/kg kefir	0
l0 mg/kg kefir	0
5 mg/kg kefir + 150 CTX	1^b^
10 mg/kg kefir + 150 CTX	1

**Score **^**a**^**0:** no change **Score **^**b**^**1:** slight change **Score **^**c**^**2:** moderate change.

**Figure 1:**
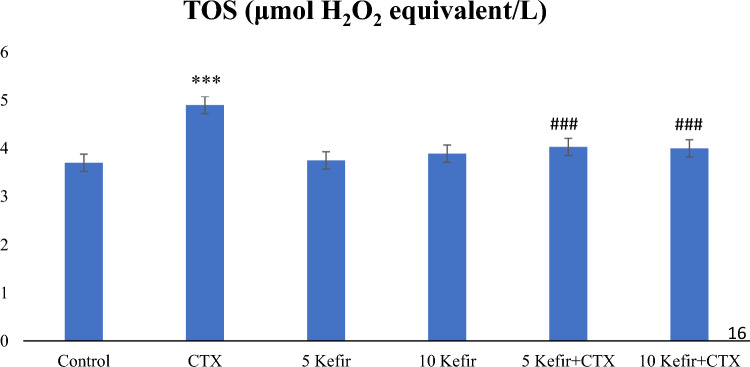
Comparison of TOS values of experimental groups administered Control, 150 mg/kg CTX, 5 mg/kg kefir, 5 mg/kg kefir + 150 mg/kg CTX, l0 mg/kg kefir, l0 mg/kg kefir + 150 mg/kg CTX. (***p < 0.001 compared to control; ###*p* < 0.001 compared to CTX group).

Total oxidant status (TOS) level, one of the parameters we measured as an indicator of CTX-induced oxidative damage, was found to be very high in the group given a single dose of 150 mg/kg CTX. TOS levels decreased significantly in the groups given 5 and 10 mg/kg kefir along with CTX. As a matter of fact, our findings showed that the TOS level, which increases with oxidative stress caused by CTX, can mostly be eliminated by kefir (Fig. 1).

**Figure 2:**
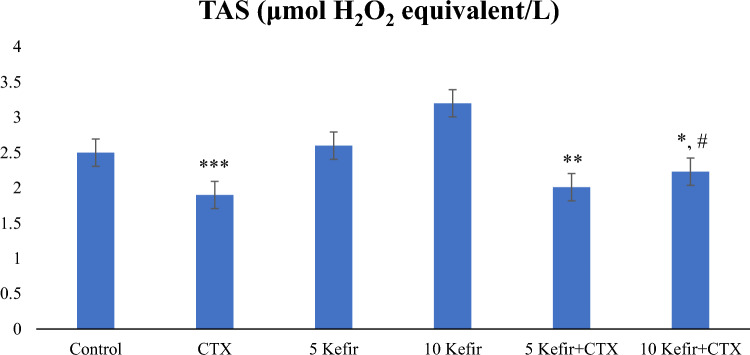
Comparison of TAS values of experimental groups administered Control, 150 mg/kg CTX, 5 mg/kg kefir, 5 mg/kg kefir + 150 mg/kg CTX, l0 mg/kg kefir, l0 mg/kg kefir + 150 mg/kg CTX. (***p < 0.001 compared to control; **p < 0.05 compared to control; *p < 0.01 compared to control; #*p* < 0.01 compared to CTX group).

Comparing the total antioxidant status (TAS) level, which is an important biomarker; In the second group, which was given only CTX, the TAS level decreased significantly. This shows that CTX causes an increase in oxidative stress and has a decreasing effect on antioxidant levels. In the groups given kefir along with CTX, the TAS level increased despite CTX and approached the control level, indicating that kefir has an antioxidative and protective effect (Fig. 2).

**Figure 3:**
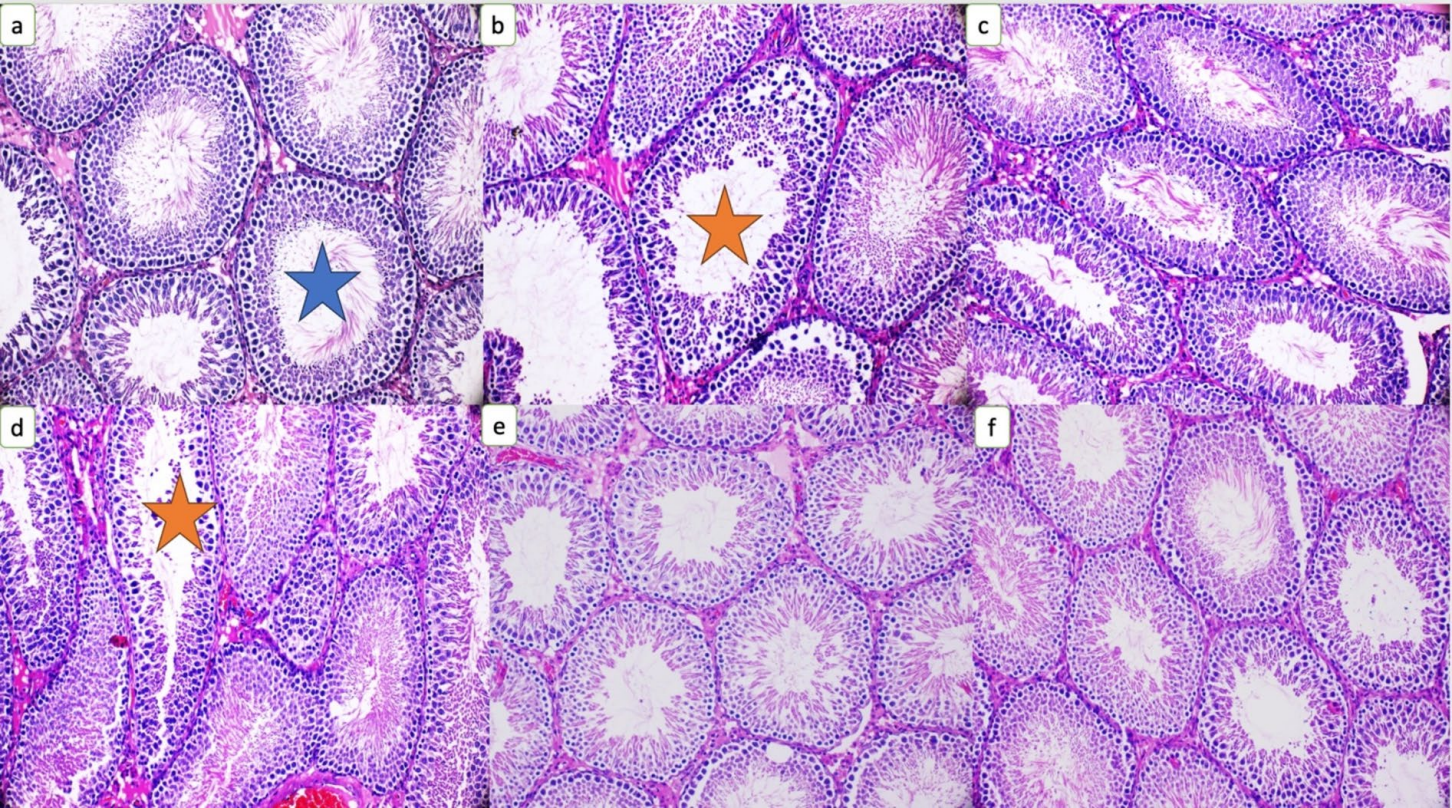
(**a**) Normal appearance tubule lumens in the transverse section of the testicle (blue star), (**b**) A tubule with impaired maturation, with no spermatids observed in the transverse section of the testicle (yellow star), (**c**) Close to normal tubule structures and tubule lumens, (**d**) A tubule with impaired maturation, with no spermatids observed in the transverse section of the testicle (yellow star), (**e**) Close to normal tubule structures and tubule lumens, (**f**) Close to normal tubule structures and tubule lumens (H&E; X200).

According to testicular histopathology findings, normal tubule lumens (blue star) were seen in the transverse section of the testis of control group animals. In the transverse section of the testicles of the group given 150 mg/kg CTX, maturated tubules (yellow asterisks) with no spermatids were observed. Close to normal tubule structures and tubule lumens were observed in rats given 5 and 10 mg/kg kefir. In the group given CTX + 10 mg/kg kefir, a maturated tubule (yellow star) with no spermatids was observed in the transverse section of the testicle. The histopathological findings of the group given 150 mg/kg CTX + 10 mg/kg kefir were better than the group given 150 mg/kg CTX + 5 mg/kg kefir (Fig. 3).

## Discussion

The most commonly used alkylating type antineoplastic drugs are used in chemotherapy to regress or stop tumor progression. Although chemotherapy basically aims to stop or destroy tumor growth without damaging healthy cells, antineoplastic drugs have low selective properties and although they destroy cancer cells, they can also cause undesirable toxicities on healthy cells. In order to enable Cyclophosphamide (CTX), an antineoplastic drug, to be used more effectively and safely in high doses, studies on the development of methods that prevent its toxic effects are important. Infertility is a major concern for patients receiving CTX therapy. In the testis, cells in the seminiferous tubules of the germinal epithelium are the most sensitive structures to the toxic effects of CTX because they have the highest mitotic and meiotic indices.

Cancer chemotherapy can cause many side effects such as infertility by causing temporary or long-term gonadal damage. According to our data, moderate changes were observed in testicular spermatid density, giant cell formation, shedding of cells into tubules, maturation disorder, and atrophy levels in the group given 150 mg/kg CTX (Table 1). In a study, it was reported that although there was no major distortion in the testicles in the CTX group, degeneration, bleeding, and cell loss were observed in the seminiferous tubules of the testicles^7^. Parallel with our study we also stated that CTX-induced reproductive damage can be attributed to oxidative stress and DNA damage^9^. Another factor that may cause oxidative stress in the testicles is defined as infection in the literature^12^. In their study, Kim et al. (2016) also determined shedding, vacuolization, decrease in the number of spermatocytes, and degeneration in the testicular germ cell epithelium of rats given CTX^13^. Likewise, in another study, significant damage such as hemorrhage between the seminiferous tubules, disorganization and separation of cells of the spermatogenic series, and vacuoles in germ cells were detected in the testicles of rats given CTX^14^. In the groups given kefir along with CTX, the moderate change seen in the CTX group decreased to a slight change and approached the control group (Table 1). In this sense, in addition to its antioxidant and antitumor properties, kefir also has an anti-inflammatory effect, suggesting that it can eliminate CTX-induced testicular damage due to oxidative stress.

Normally, the oxidative state is in balance with ROS production and ROS elimination in the cell, while disruption of this balance results in damage to the cell^15^. Some reports showed that CTX could disrupt the redox equilibrium of tissues, which suggests that the biochemical and physiological disturbances may result from oxidative stress^8^. In parallel with this information in our study, TOS level, which is an indicator of CTX-induced oxidative damage, was found to be quite high in the group given a single dose of 150 mg/kg CTX (Fig. 1). Studies showed that CTX had the lowest Johnsen score mean, consistent with its gonadotoxic effects^16,17^. Since both spermatogenesis and Leydig cell steroidogenesis are sensitive to oxidative stress, it is thought that the low oxygen tension that characterizes this tissue may be an important component of the testicles' protective mechanisms from damage caused by free radicals^6^. According to our findings, TOS levels decreased in the groups given 5 and 10 mg/kg kefir along with CTX (Fig. 1). Since these undesirable effects of CTX may be due to inducing oxidative stress in tissues and disrupting the oxidant-antioxidant balance^18^, and it has been reported that the expression of antioxidant enzymes is inhibited by CTX treatment, which reduces intratesticular testosterone concentration^19^, we aimed to use kefir, which has antioxidant properties. As a matter of fact, our results showed that kefir alleviated the oxidative damage caused by CTX by reducing the TOS level with its antioxidative properties (Fig. 1).

Disruption of the balance between antioxidant and oxidant systems causes toxicities and tissue damage. The toxic effect of CTX is related to its active metabolite, ACR. It has been stated that this toxic effect of CTX occurs by destroying the antioxidant defense systems of acrolein, which is formed as a result of its metabolism, and causes the formation of high amounts of free radicals. In our study, when we compared the TAS level, which is an important biomarker, it was seen that the TAS level decreased significantly in the only dose CTX-given group. Studies have shown that oxidative stress increases with a decrease in antioxidant enzymes^20^ and an increase in lipid peroxidation in rats treated with CTX^21^. It is reported that deterioration of the balance between antioxidant and oxidant systems causes tissue damage^22^. Antioxidative biological compounds may protect cells and tissues from the harmful effects of ROS and other free radicals produced during CTX exposure. As a matter of fact, our results show that kefir has an antioxidative and protective effect by increasing the TAS level despite CTX and approaching the control level in the groups where kefir was given together with CTX (Fig. 2). In a study, it was found that the oxidative stress index (OSI) value, which shows the status of oxidative and antioxidative systems, was higher in the CTX group than in the control group^23^.

According to testicular histopathology findings, in the transverse section of the testicles of the group given 150 mg/kg CTX, maturated tubules with no spermatids were observed (Fig. 3) and this showed that CTX damaged testicular tissue. A study's histological analysis showed that the CTX-treated group's spermatogenetic cells were disorganized and their seminiferous tubules were irregular. Moreover, spermatogenetic cells were shown to pour into the tubular lumen in this investigation, resulting in a decrease in tubule diameter^23^. In accordance with similar studies^24,25^, signs of degeneration such as atrophy in seminiferous tubules and decrease in tubule diameter, and loss of spermatogenic cells were observed in our study. Also, a decrease in tubule thickness and loss at the spermatogenic level was reflected in the sperm count and morphology of our CTX-given group findings. In a study localization and morphology of telocytes have been demonstrated in the rat male reproductive system^25^. Moreover, studies have shown that CTX, which induces oxidative stress in testicular and epididymal tissues, can cause a decrease in sperm count and motility. Additionally, it has been shown that oxidative stress caused by CTX can lead to apoptosis and shrinkage in seminiferous tubules, thinned seminiferous epithelium, and a decrease in interstitial cells and spermatogenic cells, especially in post-meiotic stages^26,27^. Testis blood barrier damage and aberrant expression of functional proteins were seen in an animal experiment with CTX intervention, wherein Sertoli cells experienced morphological, and functional abnormalities^28^. Fermented kefir, which has antioxidant, anti-apoptotic, anti-lipid peroxidation and anti-inflammatory activities^11^, could alleviate or avoid this damage. In the group given CTX + 10 mg/kg kefir, a maturated tubule with no spermatids was observed in the transverse section of the testicle. The histopathological findings of the group given 150 mg/kg CTX + 10 mg/kg kefir were better than the group given 150 mg/kg CTX + 5 mg/kg kefir (Fig. 3). We have previously shown that immune activities have been observed in humans and various animals after ingestion of lactic acid bacteria found in kefir, and it has been observed that lactic acid bacteria increase non-specific resistance against tumors or infections in humans or animals or have a strengthening effect on specific immune reactions^11^. In other studies, it has been reported that kefir consumption has antioxidant, and anticarcinogenic effects^29–31^, in parallel with the results of our study. In this study, it was observed that kefir was histologically and biochemically effective against the toxic effects of CTX on the testicles when high doses were required. For this reason, it can be stated that kefir can be used as an alternative supplement when CTX is used in high doses.

## Conclusion

The use of complementary and alternative treatments, which prevent the toxic effects of many antineoplastic chemical agents such as CTX and allow them to be used in higher or effective doses for a long time, has increased rapidly recently and has gained importance in many areas including the medical industry, the country's economy, and even social psychology. The most severe histopathological, and biochemical picture was seen in the group where high-dose CTX was used, confirming that CTX has a highly toxic effect on the testicles. An important way to ensure the effectiveness of cancer treatment is to regulate the microbiota through probiotic consumption. So, kefir is a very effective agent both in reducing the side effects of CTX and in providing cancer immunotherapy that uses the power of the patient's own immune system to destroy cancerous cells. In addition, we hope that our study will contribute to the literature for future scientific studies.

## Methods

### Kefir fermentation

In our study, commercially supplied and freeze-dried kefir yeast and 1 L of cow’s milk were preferred for kefir fermentation. Three groups of kefirs were created, with fermentation at 24–26 °C temperature at intervals of 24, 48, and 72 h on days 1, 2, and 3. It was kept at + 4 °C ready for use. We gave kefir to rats by gavage method for 12 days. Kefirs from the 1st, 2nd, and 3rd days were mixed and given by gavage method for 12 days.

### Chemicals and injections

Cyclophosphamide (CTX) (Sigma-Aldrich) was commercially available. 500 mg CTX was dissolved in 25 ml bidistilled water to prepare for injection of 150 mg/kg CTX. The injection was performed as a single dose intraperitoneally (i.p.)/body-weight (b.w.) on the 12th day of the experiment, using sterile disposable syringes.

### Ethical approval

This experimental study was approved by the *Ethics Committee of Eskisehir Osmangazi University Animal Experiments Local Ethics Committee (784-145 / 2020. And the entire study was conducted in accordance with the Animal Experiments Local Ethics Committee Directive.*

### Experimental setup

In our experimental study, healthy, males, 200 ± 20 gr, about 3 months age Wistar albino rats were used. During the experiment, the animals were kept in rooms with 12;12 light/dark lighting, 45–50% humidity, and 22 ± 2 °C temperature. And were given tap water and normal pellet feed. The 42 rats used in this study were divided into 6 groups, each group including 7 rats. Group 1 (control), single dose of 150 mg/kg/b.w CTX to the 2nd group, 5 mg/kg/b.w kefir to the 3rd group, 5 mg/kg/b.w kefir + 150 mg/kg/b.w CTX to the 4th group, 10 mg/kg/b.w kefir was given to the 5th group, 10 mg/kg/b.w kefir + 150 mg/kg/b.w CTX was given to the 6th group. Kefir was given to rats by gavage method for 12 days. A single dose of CTX was given i.p. on the last day of the experiment, namely the 12th day. At the end of the experiment, biochemical parameters and testicular tissues were taken under anesthesia.

### Biochemical parameters

#### Total antioxidant status (TAS) (mmol/L)

TAS levels were measured using commercially available kits (Relassay, Turkey). The novel automated method is based on the bleaching of the characteristic color of a more stable ABTS (2,2′-Azino-bis(3-ethylbenzothiazoline-6-sulfonic acid)) radical cation by antioxidants. The assay has excellent precision values, which are lower than 3%. The results were expressed as mmol Trolox equivalent/L.

#### Total oxidant status (TOS) (µmol/L)

TOS levels were measured using commercially available kits (Relassay, Turkey). In the new method, oxidants present in the sample oxidized the ferrous ion-o-dianisidine complex to the ferric ion. The oxidation reaction was enhanced by glycerol molecules abundantly present in the reaction medium. The ferric ion produced a colored complex with xylenol orange in an acidic medium. The color intensity, which could be measured spectrophotometrically, was related to the total amount of oxidant molecules present in the sample. The assay was calibrated with hydrogen peroxide and the results were expressed in terms of micromolar hydrogen peroxide equivalent per liter (μmol H2O2 equivalent/L).

### Histopathology

Before being examined under a light microscope, tissue samples were preserved in a 10% Neutral Buffer formaldehyde solution. Following identification, tissue samples were put into cassettes and given a two-hour rinse under running water. Tissues were run through a succession of increasing alcohol concentrations (60–100%) in order to extract water. The tissues were then polished by passing them through xylene before being implanted in melted paraffin. For each group, 4-micron-thick slices were cut from paraffin blocks and stained with hematoxylin–eosin stain. Using the Leica Q Vin 3 program on the Leica DCM 4000 computer-aided imaging system (Germany), the sections were assessed and captured on camera. A criteria table was created as a result of the evaluations made with Hematoxylin–Eosin (H&E) staining.

### Statistics

The quantitative values we obtained at the end of the study were evaluated by applying the Duncan test after one-way ANOVA, which is used in the statistical analysis of more than two independent groups, with the SPSS 26.00 statistical data program.

## Data Availability

The authors declare that all data supporting the findings of this study are available within the paper. Moreover, the datasets used and/or analysed during the current study available from the corresponding author on reasonable request.
